# Insights Into Subacute Combined Degeneration of the Cord: ‘Rabbit Ear Sign’ as a Radiodiagnostic Clue in Case of Vitamin B12 Deficiency Associated With Schizophrenia

**DOI:** 10.1155/crnm/5583285

**Published:** 2026-05-31

**Authors:** Muhammad Husnain, Muhammad Hasnat, Ahmad Nawaz, Sana Iqbal, Ahsan Numan, Muhammad Ahmad Malik, Wisha Komal, Noor Sehar, Rabia Nawaz, Talha Ali, Izhad Farooq

**Affiliations:** ^1^ Department of Neurology, King Edward Medical University, Lahore, Pakistan, kemu.edu.pk; ^2^ Department of Neurology, King Edward Medical University, Lahore, Pakistan, kemu.edu.pk; ^3^ Department of Medicine, King Edward Medical University, Lahore, Pakistan, kemu.edu.pk; ^4^ Islamic International Medical College, Islamabad, Pakistan

**Keywords:** functional vitamin B12 deficiency, homocysteine, inverted V sign, rabbit ear sign, schizophrenia, subacute combined degeneration of the cord (SCD), vitamin B12

## Abstract

**Background:**

Subacute combined degeneration manifests as the degeneration of the dorsal and lateral columns of the spinal cord. Typical symptoms include sensory deficits, weakness, ataxia, and disturbances in gait which can progress to spasticity and paraplegia. Prolonged vitamin B12 deficiency can result in irreversible neurological damage and psychiatric manifestations such as depression, irritability, hallucinations, dementia, and delirium in the affected individuals. To date, only a few cases of schizophrenia with vitamin B12 deficiency with SCD have been reported in the literature.

**Objective:**

To report a case of vitamin B12 deficiency with neuropsychiatric manifestations and rabbit ear sign as a radiological clue supporting subacute combined degeneration of the cord in vitamin B12 deficiency.

**Case Description:**

A 44‐year‐old male, who is a smoker and was diagnosed with schizophrenia 18 months ago, presented with a gradual, symmetrical, and painless weakness affecting all four limbs over the past 6 months. Neurological examination revealed spastic quadriparesis with impaired dorsal column. Laboratory tests were suggestive of mild to moderate anemia with a peripheral blood smear showing macrocytosis. Neuroimaging of the craniocervical junction revealed a characteristic “rabbit ear sign” or “inverted V sign”. Although serum vitamin B12 and folate levels were within normal range, homocysteine levels were significantly elevated, leading to a diagnosis of subacute combined degeneration (SCD) of the spinal cord secondary to vitamin B12 deficiency. Methylmalonic acid testing was unavailable locally; however, marked hyperhomocysteinemia (> 15 μmol/L) with neuropsychiatric manifestations and anemia strongly supports functional vitamin B12 deficiency, as neurological features may occur in the absence of overt macrocytosis. Vitamin B12 replacement resulted in marked improvement in functional status of the patient, in terms of spasticity, as well as, motor, sensory and psychiatric symptoms.

**Conclusion:**

This case highlights the need to evaluate vitamin B12 deficiency in young patients with neuropsychiatric symptoms, even with mild to moderate anemia, as early replacement can prevent irreversible neurological damage from SCD.

## 1. Introduction

Subacute combined degeneration (SCD) is an acquired myelopathy which is caused by the spinal affliction of vitamin B12 deficiency, nitrous oxide (NO), and certain medications [1, 2] which manifests as the degeneration of the dorsal and lateral columns of the spinal cord. SCD commonly affects the posterolateral columns of spinal cord including the dorsal column and lateral columns and rarely the pyramidal and spinocerebellar tracts [3]. The earliest sign of vitamin B12 deficiency is demyelination in the posterior columns of the thoracic spinal cord. This stage is also regarded as the most reversible phase of pathology in SCD [4]. Clinical characteristics of SCD include disturbance of position sense, ascending paresthesia, ataxia, impaired gait and paraparesis [3]. Psychiatric symptoms such as depression, irritability, hallucinations, dementia, and delirium may also be present in the affected individuals [5]. It may result from insufficient dietary intake, pernicious anemia or exposure to NO during anesthesia.

Vitamin B12 deficiency is associated with a range of symptoms encompassing hematological, neurological, psychological, and neuropsychological disorders. It may arise from insufficient dietary intake or inadequate absorption in the gut. Regardless of the cause, prolonged vitamin B12 deficiency can result in irreversible neurological damage and psychiatric manifestations in affected individuals.

SCD can be diagnosed using MRI, but in 60%–85% of cases, MRI scans of the spine appear normal. When abnormalities are detected, they typically present as symmetric bilateral high signal areas within the dorsal columns, forming a distinct pattern known as the inverted “V” sign [6].

Given the significant clinical implications of delayed or missed diagnosis, there is a pressing need to improve diagnostic accuracy through a better understanding of MRI findings in SCD. This study aims to explore the prevalence and diagnostic significance of the inverted “V” sign in patients with SCD, enhancing the overall diagnostic process for this condition, especially guiding the physician and the radiologist to make an early diagnosis on the basis of physical examination and radiodiagnosis, to improve the overall outcome of patients with vitamin B12 deficiency and SCD.

## 2. Case Description

A 44‐year‐old male from Lahore, Pakistan, a known case of schizophrenia for the last 18 months, presented with complaints of gradual, symmetrical, progressive, and painless quadriparesis for the last 6 months. The patient was a known case of schizophrenia due to his symptoms of audiovisual hallucinations, irritability, delusions, and anger. He was already taking antipsychotics (haloperidol) with poor compliance in the last 18 months, 6 months back. Initially, he reported difficulty buttoning his shirt and climbing the stairs which worsened further and it resulted in difficulty holding objects and eventually the patient was unable to walk with support for the last 2 months. He had a history of blood transfusion 2 times in the last 6 months due to anemia and a positive sexual history for unprotected intercourse 2 years back, although the history of drug intake or any substance abuse was unremarkable.

On examination, there was no clubbing, paronychia, or koilonychia. There was a contracture on the left hand. No conjunctival pallor was seen. There was marked quadriparesis with a power of 4/5 in lower limbs and a power of 4/5 in upper limbs (MRC scale). There was increased tone in all limbs of Grade 4 on the Ashworth scale. There was also bilateral impairment of joint position sensation and mild sensory deficit. There was hyperreflexia in both upper and lower limbs, and plantar reflexes were upgoing bilaterally.

His labs showed normal liver function tests, kidney function tests, and normal serum electrolytes. His complete blood count showed Hb of 10.1 with a mean corpuscular volume (MCV) of 94. Other abnormal lab findings were high lactate dehydrogenase (LDH) levels of 799 U/L. His other labs, i.e., stool analysis, urine complete exam, HIV, hepatitis B and C screening, rheumatoid factor, anticyclic citrullinated peptide (anti‐CCP), antinuclear antibody (ANA) profile, were all within normal range. His cerebrospinal fluid analysis, abdominal ultrasound and computed tomography of the chest, abdomen, and pelvis were also normal. There was no history of NO exposure; however, any history of recreational exposure of NO could not be ruled out.

**FIGURE 1:**
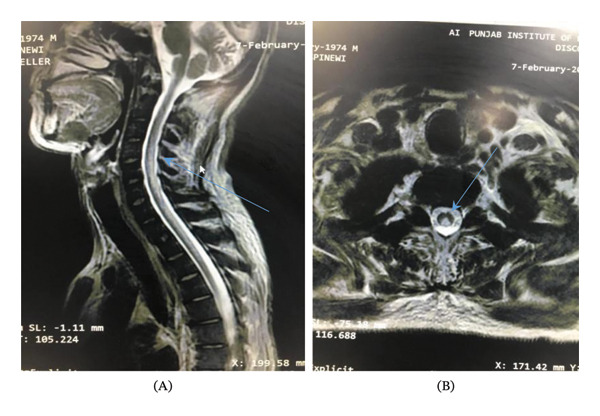
(A) T2 weighted image of sagittal section of dorsal cervical spine showing hyper intensity in cervical cord C1‐C6. (B) Inverted V sign in axial view T1 weighted image.

**FIGURE 2:**
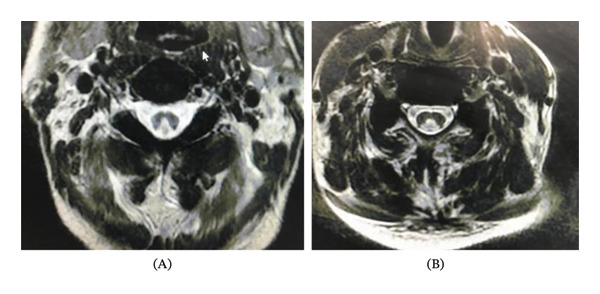
(A and B) T2 and T1 weighted images showing inverted V or rabbit ear sign in the cervical cord.

A complete blood count with peripheral smear was performed which showed anisopoikilocytosis, macrocytosis, target cells and a few pencil shaped cells. Neutrophils were of normal morphology. His vitamin B12 and serum folate levels were normal but his homocysteine levels showed a high level of 18.93 μmol/L. Serum methylmalonic acid (MMA) levels, although more specific in B12 deficiency, were not conducted due to local unavailability. Although SCD typically involves the posterior columns of the cervical and thoracic cord, craniocervical junction MRI was prioritized due to prominent neuropsychiatric symptoms and the possibility that early cervical lesions may produce significant clinical manifestations before thoracic involvement becomes evident. MRI craniocervical junction showed a hyperintensity throughout the cervical cord in T1 and T2 weighted axial images (Figure 1 and 2).

The differential diagnoses included SCD due to vitamin B12 deficiency, degenerative cervical myopathy, substance abuse, and taboparesis secondary to HIV (history of unprotected sexual intercourse). After thorough investigations, a diagnosis of SCD of the spinal cord due to vitamin B12 deficiency was made, and the patient was started on aggressive vitamin B12 therapy which was parenteral vitamin cobalamin 1000 mcg once daily for 1 week, followed by once a week for 3 months and once a month afterwards. Meanwhile, folic acid was also replaced. He was also started with oral Baclofen 30 mg daily and a physical rehabilitation therapy.

**FIGURE 3:**
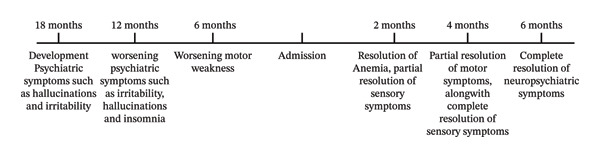
Timeline chart (CARE guidelines).

After 2 months of follow‐up, the patient reported mild improvement in sensory deficit and psychiatric symptoms; however, his motor weakness and hyperreflexia persisted. His spasticity had improved significantly (Ashworth score 2). The patient’s anemia improved, and his Hb levels were 11.6 after 2 months. Moreover, his homocysteine levels were also within normal range after a follow‐up of 3 months. After 4 months, his motor weakness had also improved, and the patient was able to walk on his own (Figure 3).

The cause of vitamin B12 deficiency was not linked to a malabsorption syndrome, as there was no history of chronic diarrhea and intrinsic factor antibodies were normal. The deficiency was primarily due to decreased intake of a cobalamin‐rich diet (see Figure 3).

## 3. Discussion

Vitamin B12 is a crucial water‐soluble vitamin essential for cellular metabolism and maintaining the integrity of the nervous system [5]. Its neurological and psychiatric complications are rare but are generally caused by persistent and severe vitamin B12 deficiency for a very long time. The primary neurological symptoms include paraesthesia, ataxia, and limb weakness [7]. The psychiatric manifestations can be attributed to the role of vitamin B12 in the synthesis of neurotransmitters. Its deficiency can lead to altered monoamine synthesis and elevated homocysteine levels, both of which have been implicated in psychiatric disorders. The exact mechanisms remain complex and multifactorial, involving metabolic, genetic, and environmental factors. Prolonged vitamin B12 deficiency can cause SCD which involves the degeneration of the dorsal (posterior) and lateral columns of the spinal cord, which are responsible for proprioception, vibration sensation, and motor function. Vitamin B12 is essential for myelin synthesis, and its deficiency disrupts this process, leading to demyelination and subsequent neuronal damage [8].

Vitamin B12 deficiency can have multiple underlying causes, making it challenging to pinpoint the exact origin of the deficiency. Essential diagnostic tools for identifying vitamin B12 deficiency include basic laboratory tests, such as a full blood count (FBC) and peripheral blood smear analysis, as illustrated in our case [9]. MRI scans of the spine can appear normal in 60%–85% of cases. When abnormalities are detected, they typically present as symmetric bilateral high signal areas within the dorsal columns. This distinct pattern is often referred to as the inverted “V” sign [6].

In roughly 20% of patients displaying neurological signs and symptoms, there’s a chance that within 3 months of initiating therapy, some degree of recovery might become apparent, albeit only partially [10]. The time required to alleviate symptoms of vitamin B12 deficiency can differ. If there’s no clinical improvement after 4–8 weeks for anemia or after 6–12 months for neurological signs, it could indicate that the symptoms are not attributable to B12 deficiency or that the dosage or method of administering vitamin B12 needs adjustment based on symptom severity [11]. The duration required for the symptoms to resolve is directly related to the degree of severity and the duration of the disease [1]. Additionally, full resolution of the symptoms is achievable only if they have been present for just a few weeks prior to beginning treatment [12].

The case highlights several important considerations for clinical practice. Firstly, it emphasizes the need for heightened awareness of vitamin B12 deficiency as a potential underlying cause of neuropsychiatric symptoms. Given its treatable nature, early diagnosis and intervention can prevent significant morbidity. Secondly, the case illustrates the importance of a multidisciplinary approach in managing patients with complex presentations. Neurologists, psychiatrists, and primary care physicians must collaborate to ensure comprehensive care, addressing both the neurological and psychiatric aspects of the condition.

There were a few limitations in this case, such as we were unable to investigate the MMA levels in this patient due to its unavailability in our region. The patient refused to undergo a follow‐up MRI to see if the MRI findings were also reversed along with the symptoms. Additionally, the patient refused to have his bone marrow biopsy done.

## 4. Conclusion

The clinical significance of this case report is so paramount as a young patient with neuropsychiatric symptoms predominantly schizophrenia with pre‐existing mild‐to‐moderate anemia needs workup for vitamin B12 deficiency and follow‐up for SCD. Assessment of MMA and homocysteine enables early detection, with MRI findings further improve diagnostic accuracy. Since prolonged deficiency of vitamin B12 can lead to irreversible neurological damage, prompt replacement therapy should be initiated to prevent such neurological complications.

## Funding

No funding was received for this manuscript.

## Consent

Written informed consent was obtained from the patient for publication of this case report and accompanying clinical details/images.

## Conflicts of Interest

The authors declare no conflicts of interest.

## Data Availability

The data that support the findings of this study are available from the corresponding author upon reasonable request.
